# Self-Controlled Feedback and Behavioral Outcomes in Motor Skill Learning: A Meta-Analysis

**DOI:** 10.3390/bs15091291

**Published:** 2025-09-22

**Authors:** Biye Wang, Tao Tao, Yuchen Yuan, Wei Guo

**Affiliations:** College of Physical Education, Yangzhou University, Yangzhou 225000, China; wangbiye@yzu.edu.cn (B.W.) mx120240506@stu.yzu.edu.cn (T.T.); mz120210786@stu.yzu.edu.cn (Y.Y.)

**Keywords:** motor learning, augmented feedback, self-controlled feedback, meta-analysis

## Abstract

Providing feedback is a key instructional strategy in motor learning. Recently, interest has grown in self-controlled (SC) feedback, which allows learners to choose when to receive feedback. However, evidence on its effectiveness remains mixed, and its impact across different learning phases and populations is unclear. This meta-analysis assessed the effect of SC feedback on motor skill learning during acquisition, retention, and transfer phases, compared to passively received (PR) feedback and Yoked (YK) feedback groups. A comprehensive search of three datasets identified 29 studies comprising 1147 participants. Although SC feedback did not bring a significant advantage in the acquisition phase, it facilitated motor skill learning in both the retention and transfer phases. Moderator analyses revealed that cognitive status influenced SC feedback efficacy during the acquisition phase, with significant effects observed only in cognitively impaired individuals. Additionally, skill type moderated outcomes in the transfer phase, with series skills showing greater benefits than discrete skills, and this effect was significant only when compared to the YK group. In summary, SC feedback-enhanced motor skill learning in the retention and transfer phases, supporting its role in the consolidation and generalization of learning. Additionally, cognitively impaired individuals showed greater benefits during the acquisition phase, and series skills demonstrated more substantial gains in the transfer phase.

## 1. Introduction

Augmented feedback, defined as performance-related information from an external source that supplements sensory input (Moinuddin et al., 2021; Newell, 1976; Winstein & Schmidt, 1990), is a critical factor in motor skill learning, in conjunction with the amount of practice (Newell, 1976; Yadi, 2016). In most studies, augmented feedback has been provided as passively received (PR) information about motor skill performance, with the timing and frequency of feedback determined by the experimenter or another external source (Hermassi et al., 2019; Hicheur et al., 2020; Puklavec et al., 2021; Yamamoto et al., 2022). Recent research has indicated that providing learners with control over feedback, known as self-controlled (SC) feedback, can enhance motor skill learning (Carter et al., 2014; Chiviacowsky, 2014; van der Meer et al., 2024).

SC feedback allows learners to control and determine the timing of feedback (Carter et al., 2014; Carter & Patterson, 2012; Chiviacowsky, 2014; Chiviacowsky & Wulf, 2002). Typically, studies comparing SC to PR feedback evaluate separate groups learning the same motor skill tasks (Hansen et al., 2011; Hebert & Coker, 2021). In these studies, SC groups control their feedback schedule autonomously, determining when to receive feedback. In contrast, the PR feedback schedule is controlled by researchers, coaches, peers, or others, thereby leaving learners unable to choose and only passively receiving feedback. Yoked (YK) feedback, a specialized PR feedback technique that features strict experimental control, is used as a comparator in numerous studies on SC feedback. As in PR, the timing of feedback in YK is not determined by the learner. However, unlike conventional PR, YK feedback schedules are synchronized with those of SC groups, ensuring identical temporal delivery of feedback. This method ensures that feedback characteristics are identical between the SC and YK groups, allowing the attribution of observed differences to the control of feedback rather than the type of feedback.

Janelle et al. (1995) were the first to test the effect of SC feedback on motor skill learning, and they demonstrated its benefit. SC feedback improves motor learning of several tasks, which include serial (Huet et al., 2009; Patterson & Carter, 2010; Woodard & Fairbrother, 2020) and discrete motor skill learning (Grand et al., 2015; Hebert & Coker, 2021; Souissi et al., 2021), and it enhances motor learning not only in healthy adults (Chiviacowsky, 2014; Couvillion et al., 2019; Hebert & Coker, 2021; Kaefer et al., 2014; Patterson et al., 2019; Post et al., 2016), but also in children (Chiviacowsky et al., 2008; Hemayattalab et al., 2013), elderly persons (Kwon et al., 2014), and patients with Parkinson’s disease (Chiviacowsky et al., 2012b) and cerebral palsy (Hemayattalab & Rostami, 2010).

However, it should be noted that some studies failed to replicate the benefits of SC feedback (Bacelar et al., 2022; McKay & Ste-Marie, 2020; McRae et al., 2015; St Germain et al., 2022). For example, learning the non-dominant arm bean bag toss task was not significantly improved by SC compared to YK feedback (Bacelar et al., 2022). In a serial-timing task, delayed retention test results showed a lower variable error for learners with PR compared to SC feedback (McRae et al., 2015). Therefore, the influence of SC feedback on motor skill learning continues to be debated. Because meta-analysis enables the extraction of data from numerous studies to quantify the effect of SC feedback on motor skill learning and identify sources of heterogeneity through the analysis of moderating variables, it is used to discuss the aggregated results of multiple studies (Page et al., 2021).

Existing meta-analyses on SC feedback have employed varying methods and produced conflicting conclusions, indicating a need for updated evaluation. For instance, Jimenez-Diaz et al. (2020) analyzed 18 articles published up to 2019 and reported significant benefits of SC feedback in acquisition that persisted into retention. However, their analysis focused on within-group performance changes over time, rather than the more common group-by-group comparison, and did not address the transfer phase, a critical indicator of skill generalization. In contrast, McKay et al. (2022, 2023) applied advanced bias-correction methods (e.g., weight-function models, *p*-curve analysis, robust Bayesian meta-analysis, *z*-curve), which challenged earlier overly optimistic interpretations and emphasized the need for larger and better-controlled studies. Research underscores the importance of both retention and transfer tests for evaluating the permanence and generalization of learned skills (Kantak & Winstein, 2012). While some studies reported SC feedback advantages in both retention and transfer (Carter et al., 2014; Hansen et al., 2011; van Maarseveen et al., 2018), others provided evidence for benefits only in transfer (Carter et al., 2017; Magill & Hall, 1990). Recent meta-analyses were last updated in 2019, excluding more recent studies (Bacelar et al., 2022; St Germain et al., 2022; van der Meer et al., 2024; Yantha et al., 2021). Moreover, although comparability was ensured by inclusion criteria, the aggregated analyses combined heterogeneous task types and participant populations with limited exploration of condition-specific effects, and some included experiments exhibited methodological inconsistencies such as imperfectly matched controls. These issues, alongside the publication and reporting biases already identified, highlight the need for updated meta-analytic work that incorporates recent studies, applies more nuanced subgroup analyses, and considers a broader range of outcomes.

Furthermore, the aforementioned meta-analysis concentrated on the effectiveness of SC feedback in healthy participants, neglecting factors such as the cognitive states of special populations, which are intrinsically linked to real-life motor skill learning. Individuals with cognitive impairments often exhibit deficits in motor proficiency, including posture, gross motor skills, and fine motor skills (Alesi & Battaglia, 2019; David et al., 2012; Fernandes et al., 2024; McQuillan et al., 2021). Fortunately, motor interventions have been shown to significantly enhance motor proficiency levels in these populations (Anderson et al., 2024; Fernandes et al., 2022), and SC feedback also holds potential to facilitate their motor skill learning. Another meta-analysis evaluated the effect of SC feedback on retention test performance measures (Hemayattalab, 2014; Pashabadi et al., 2021; Zamani et al., 2015). Although positive effects were found in published studies, they were insufficient to demonstrate that SC feedback is more effective than YK practice (McKay et al., 2022).Other moderator variables were selected based on their potential influence on motor skill learning. The environment (indoor vs. outdoor) was considered because outdoor settings provide higher ecological validity, whereas laboratory environments are more controlled and stringent, which may affect performance and learning outcomes. Feedback models likewise require further scrutiny. Verbal and visual feedback are common methods of providing SC feedback. In a double-mini trampoline task in which verbal feedback was used, skill progress in the retention test was significantly greater among recipients of SC compared to YK feedback (Ste-Marie et al., 2015). With visual feedback, young adults receiving SC feedback showed a greater learning advantage in the release slider task (Carter & Patterson, 2012). The role of feedback models on the impact of SC feedback on motor skill learning remains controversial. Mödinger et al. (2021) suggested that video-based visual feedback enhances motor learning more effectively than solely verbal feedback. Jaszczur-Nowicki et al. (2021) found that combining verbal with visual feedback proved more effective than providing either form alone. Therefore, this study also sought to clarify how cognitive states and feedback models modulate the impact of SC feedback on motor skill learning.

Consequently, we aimed to conduct a meta-analysis to assess the effects of SC feedback during the acquisition, retention, and transfer phases, while also exploring relevant moderating factors.

## 2. Materials and Methods

This study was reported in accordance with the Preferred Reporting Items for Systematic Reviews and Meta-Analysis (PRISMA) guidelines and was registered with the International Prospective Register of Systematic Reviews (PROSPERO, CRD42023401718).

### 2.1. Search Strategy

Systematic literature searches were conducted in the PubMed, Web of Science and Cochrane library through December 2024. The following keywords were used: motor terms (“motor learning” OR “motor skill” OR “skill acquisition” OR “motor performance”), AND feedback terms (“augmented feedback” OR “self-controlled feedback” OR “self-regulated” OR “learner-controlled” OR “self-control”). To find additional pertinent papers, the reference lists of the retrieved articles were examined manually. Two impartial reviewers (B.W, T.T) independently conducted the initial screening using titles and abstracts. Full-text evaluation was used to further screen the remaining articles. Consensus and discussion with another two authors (Y.Y, W.G) facilitated the resolution of any disputes between the two reviewers.

### 2.2. Eligibility Criteria

Studies were included for this meta-analysis if the following criteria were met: (1) randomized controlled trial design; (2) intervention method: self-controlled feedback was used in the intervention group but not in the control group; (3) measurements of motor performance included at least one outcome that could be used to calculate an effect size; and (4) written in the English language. The following were excluded: (1) non-interventional studies; (2) editorials or conference abstracts; (3) studies that cannot extract relevant data or datasets that could not be used in the meta-analysis due to inconsistencies or extreme values; (4) review or theoretical articles; and (5) reports not retrieved.

### 2.3. Data Extraction and Statistical Analysis

The main outcome of this study was the standardized mean difference (SMD) of motor performance of motor skills calculated from the mean and standard deviation of the acquisition, retention, and transfer phases reported in the original study. The SMD between the experimental and control groups served as a measure of the intervention effect (Maher et al., 2003). Positive SMDs indicated intervention effectiveness. The pooled SMD was computed by averaging the effect sizes of each study. Statistics were quantified and effect sizes were calculated using Comprehensive Meta-Analysis software (version 3.3). The homogeneity of interstudy effect size changes was tested based on the Q-statistic.

To evaluate the heterogeneity of the included studies, the *I*^2^-statistic was utilized. A fixed-effects model would be employed for analysis if the heterogeneity test resulted in *p* ≥ 0.05 and *I*^2^ < 50%, indicating no statistical heterogeneity between studies. A random effects model would be utilized if *p* < 0.05 and *I*^2^ ≥ 50%, indicating statistical heterogeneity between studies. Egger’s regression tests and funnel plots were employed to evaluate potential small study effects (publication bias, etc.).

In each phase, motor skill performance was determined based on the provided data. Acquisition phase performance, if not explicitly specified, was derived from the last block of acquisition data. For the retention and transfer phases, performance was calculated as the mean value of the corresponding blocks. In several studies, PR conditions included multiple groups with different feedback frequencies, and unclear selection of comparison groups could potentially bias the results. To ensure consistency and transparency, we selected the PR group with a feedback frequency most closely matching that of the SC group. When multiple eligible PR groups were present, either merging procedures or statistical adjustments were applied to avoid duplicate counting. When multiple measures were reported, we prioritized the primary outcome specified by the original authors (e.g., performance score). If no primary outcome was indicated, we selected the measure most relevant to the learning task (e.g., absolute constant errors in a key-pressing task).

### 2.4. Risk of Bias Assessment

Two reviewers assessed the included articles’ methodological quality independently. Quality was evaluated using the PEDro scale from the Physiotherapy Evidence Database (Maher et al., 2003). Before arriving at a conclusion, discordant ratings were resolved by consensus or discussion with author D. Potential quality scores for each item ranged from 0 to 10, with higher scores reflecting research of a better caliber.

## 3. Results

### 3.1. Study Characteristics

**Figure 1:**
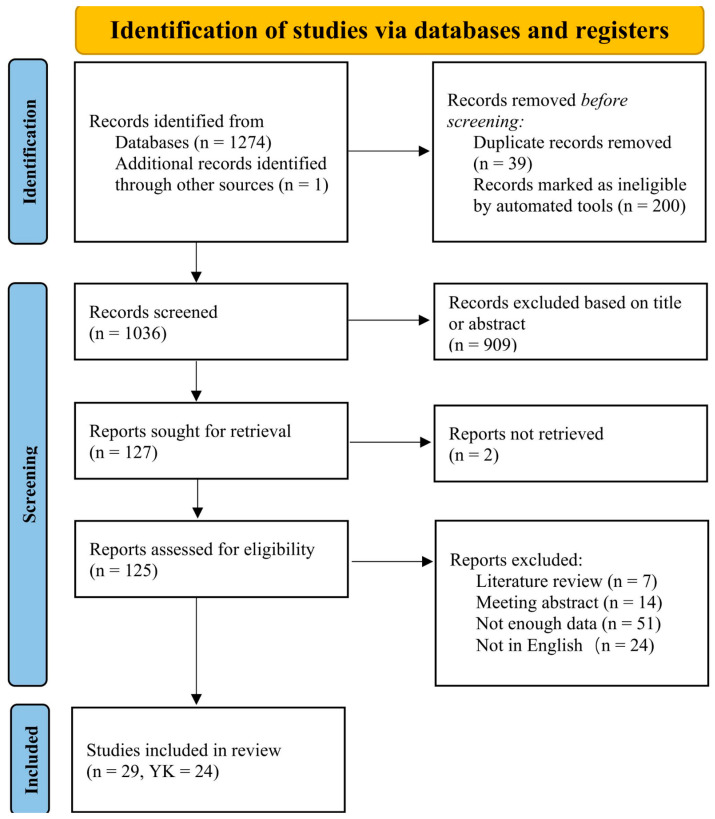
Selection process for the meta-analysis.

The meta-analysis included 29 eligible studies comprising 1147 participants in combined intervention groups, 555 in self-controlled (SC) groups, and 592 in passively received (PR) groups, including 470 in yoked (YK) groups. An overview of the selection process is provided in Figure 1. A detailed list of the studies in the “reports excluded” section and the corresponding rationale can be found in Supplementary File S1.

The mean participant age ranged from 7 to 70 years old; however, it is noteworthy that the majority of the research participants were college students. Sample sizes ranged from 12 to 152 (median sample size = 28). The main characteristics of the 29 articles are summarized in Table 1.

### 3.2. SC Versus PR Feedback in Acquisition Phase

**Figure 2:**
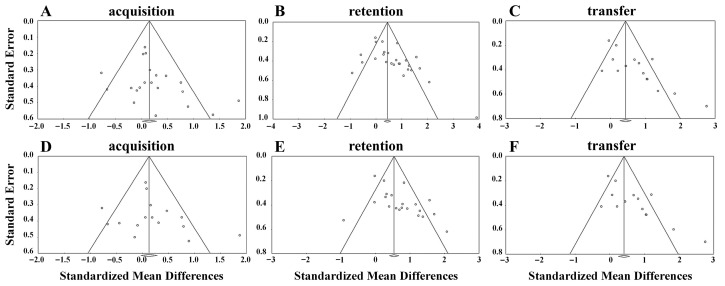
Funnel plots for the three phases. (**A**–**C**) compare SC and PR feedback, while (**D**–**F**) compare SC and YK feedback.

**Figure 3:**
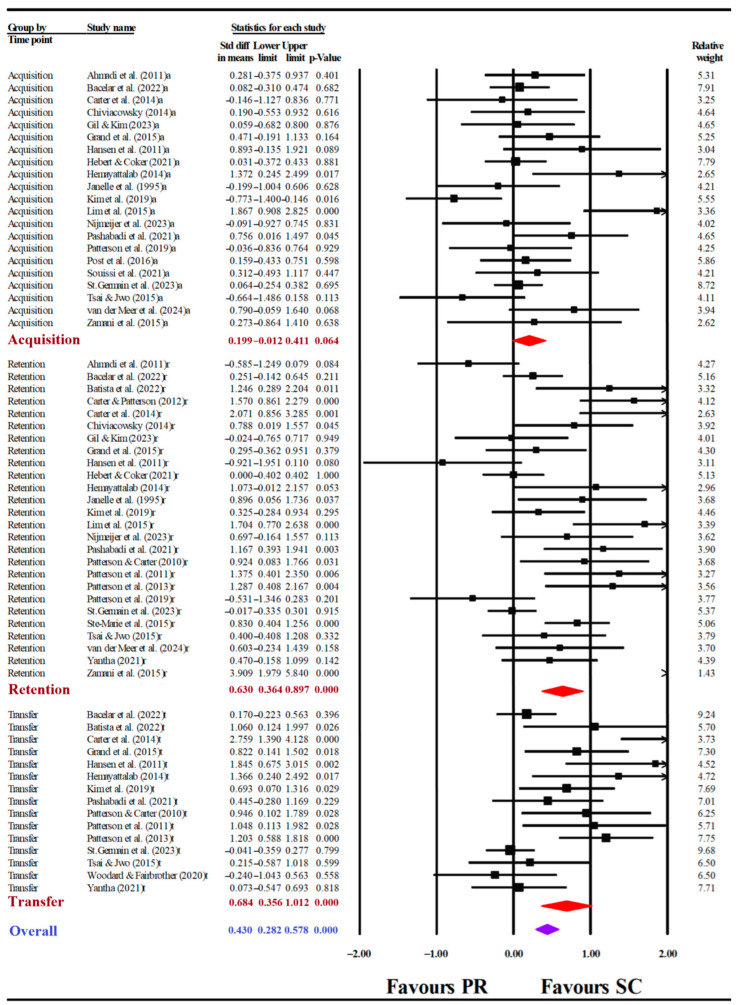
Forest plot of the self-controlled (SC) group compared to the passively received (PR) group. Black squares represent effect sizes from individual studies; red diamonds indicate the pooled effects for the acquisition (a), retention (r), and transfer (t) phases; purple diamonds indicate the overall effect across all phases.

The acquisition phase featured significant heterogeneity (Q _(20)_ = 39.353, *I*^2^ = 49.178, *p* < 0.01), prompting the use of the random effects model. To confirm the results of the sensitivity analysis, a meta-analysis was conducted by sequential removal of single studies. After removal of single studies, *I*^2^ values ranged from 45.293 to 51.717, with little difference from the overall *I*^2^ = 49.178, with the exception of two studies (Kim et al., 2019; Lim et al., 2015), which shifted *I*^2^ to 38.065 and 28.856, respectively. Overall *I*^2^ values after removal of single studies are shown in Supplementary File S2. The funnel plot did not reveal significant asymmetry (Figure 2A), and the Egger’s test did not reveal publication bias (*t* = 1.550; *df* = 19; *p* = 0.137). Consequently, the meta-analysis of the acquisition phase included 21 studies and did not exclude any. Motor learning performance did not differ between SC and PR groups (SMD = 0.199, 95% confidence interval (CI) [−0.012, 0.411], *p* = 0.064; Figure 3).

### 3.3. SC Versus PR Feedback in Retention and Transfer Phases

The retention phase exhibited significant heterogeneity (Q_(25)_ = 94.149, *I*^2^ = 73.446, *p* < 0.001), so the random effects model was used. In terms of sensitivity analysis, *I*^2^ values ranged from 70.623 to 74.506 after removal of single studies, with little difference from the overall *I*^2^ = 73.446, suggesting that all studies contributed to the stability of the overall results. The funnel plot revealed slight asymmetry (Figure 2B). The Egger’s test indicated a significant publication bias (*t* = 3.167; *df* = 24; *p* < 0.01). Sensitivity analysis revealed that the SMD fluctuated between 0.576 and 0.679 after excluding any single study, a range that encompassed the overall SMD, indicating a relatively stable effect. Consequently, the meta-analysis of retention included 26 studies.

The transfer phase also exhibited significant heterogeneity (Q_(14)_ = 46.626, *I*^2^ = 69.974, *p* < 0.001); consequently, the random effects model was used. In terms of sensitivity analysis, *I*^2^ values ranged from 62.822 to 72.118 following the removal of single studies, with little difference from overall *I*^2^ = 58.292. The funnel plot revealed significant asymmetry. (Figure 2C). The Egger’s test indicated a significant publication bias (*t* = 4.272; *df* = 13; *p* < 0.001). Sensitivity analysis showed that the SMD ranged from 0.585 to 0.755 after excluding any single study, encompassing the overall SMD and indicating a relatively stable effect. Consequently, the meta-analysis of transfer included 15 studies.

The SC groups demonstrated superior motor learning performance compared to the PR groups in both the retention (SMD = 0.630, 95%CI [0.364, 0.897], *p* < 0.001) and transfer phases (SMD = 0.684, 95%CI [0.356, 1.012], *p* < 0.001; Figure 3).

### 3.4. Moderator Analysis of SC Compared to PR Feedback

The results of the moderator analysis, including SMD values, 95% CIs, and homogeneity test statistical values, are summarized in Table 2. The following variables were analyzed: cognitive status (normal or impaired); feedback model (verbal or visual); environment (outdoor or indoor); skill type (series or discrete); and mean participant age (adult or adolescent). Skill level was not analyzed due to the small sample size (*n* < 3), and for the same reason, cognitive and environment status were not analyzed in the transfer phase.

Moderating variables had specific effects on motor skill learning outcomes during different learning phases. In the acquisition phase, significant heterogeneity was observed between the two cognitive status subgroups (Q_(1)_ = 4.895, *p* < 0.05). Specifically, SC feedback improved motor performance significantly among cognitively impaired participants (SMD = 0.789, 95% CI [0.246, 1.333], *p* < 0.01) but not among cognitively normal participants (SMD = 0.130, 95% CI [−0.082, 0.343], *p* = 0.229). Regarding the feedback model, neither verbal (SMD = 0.118, 95% CI [−0.150, 0.387], *p* = 0.388) nor visual (SMD = 0.270, 95% CI [−0.034, 0.575], *p* = 0.082) feedback exerted significant effects. Neither indoor (SMD = 0.205, 95% CI [−0.076, 0.486], *p* = 0.152), nor outdoor (SMD = 0.241, 95% CI [−0.098, 0.580], *p* = 0.163) environments brought significant effects. No significant intervention effects were observed for discrete (SMD = 0.087, 95% CI [−0.020, 0.287], *p* = 0.087) or series (SMD = 0.446, 95% CI [−0.705, 1.598], *p* = 0.448) skill types or for adolescents (SMD = 0.332, 95% CI [−0.421, 1.084], *p* = 0.388) or adults (SMD = 0.176, 95% CI [−0.030, 0.381], *p* = 0.093).

In the retention phase, significant intervention effects were observed for both normal (SMD = 0.535, 95% CI [0.275, 0.797], *p* < 0.001) and impaired (SMD = 1.768, 95% CI [0.479, 3.056], *p* < 0.01) cognitive status; verbal (SMD = 0.666, 95% CI [0.306, 1.025], *p* < 0.001) and visual (SMD = 0.598, 95% CI [0.206, 0.990], *p* < 0.01) feedback models; for both outdoor (SMD = 0.550, 95% CI [0.098, 1.002], *p* < 0.05) and indoor (SMD = 0.656, 95% CI [0.321, 0.990], *p* < 0.001) environments; for both series (SMD = 0.628, 95% CI [0.085, 1.170], *p* < 0.05) and discrete (SMD = 0.630, 95% CI [0.291, 0.968], *p* < 0.001) skill types; and for both adolescents (SMD = 1.072, 95% CI [0.430, 1.715], *p* < 0.01) and adults (SMD = 0.533, 95% CI [0.246, 0.821], *p* < 0.001).

In the transfer phase, significant intervention effects were observed for both visual (SMD = 0.941, 95% CI [0.445, 1.437], *p* < 0.001) and verbal (SMD = 0.372, 95% CI [0.009, 0.735], *p* < 0.05) feedback; for both series (SMD = 1.040, 95% CI [0.697, 1.383], *p* < 0.001) and discrete (SMD = 0.553, 95% CI [0.157, 0.948], *p* < 0.01) skill types; and for both adolescents (SMD = 0.704, 95% CI [0.269, 1.140], *p* < 0.01) and adults (SMD = 0.719, 95% CI [0.289, 1.140], *p* < 0.01).

### 3.5. SC Versus YK Feedback in Three Phases

Considering that the YK group had better control over experimental conditions since it matched the feedback schedule (number of times, order, etc.) to that of the SC group, and noting that most studies used the YK group as the control for SC, this meta-analysis included only studies comparing the SC and YK groups. The analysis focused on these groups under strict control conditions to assess differences. Consequently, two effect sizes from one study (Janelle et al., 1995) and all data from five studies (Ahmadi et al., 2011; Hebert & Coker, 2021; Hemayattalab, 2014; Patterson et al., 2019; Zamani et al., 2015) were excluded.

The three phases of acquisition (Q_(15)_ = 35.328, *I*^2^ = 57.541, *p* < 0.01), retention (Q_(20)_ = 58.795, *I*^2^ = 65.984, *p* < 0.001), and transfer (Q_(13)_ = 43.932, *I*^2^ = 70.409, *p* < 0.001) indicated significant heterogeneity; consequently, the random effects model was used. Sensitivity analyses indicated that most studies made stable contributions to the overall results (Supplementary File S2), with the exceptions of two studies in the acquisition phase (Kim et al., 2019; Lim et al., 2015) and one effect size from one study in the transfer phase (Carter et al., 2014) that caused a large change in *I*^2^ values after removal. The funnel plot exhibited symmetry similar to that observed with PR (see Figure 2D–F). Egger’s test for the acquisition phase (*t* = 0.929, *df* = 14, *p* = 0.369) indicated no significant publication bias, while significant publication bias was found in the retention (*t* = 2.863, *df* = 19, *p* < 0.01) and transfer (*t* = 3.896, *df* = 12, *p* < 0.01) phases. Sensitivity analysis revealed that, overall, SMDs remained within the fluctuation ranges of the retention (from 0.462 to 0.745) and transfer (from 0.545 to 0.722) phases after the removal of individual studies, indicating good stability. Consequently, 16 studies were included in the acquisition phase, 21 in the retention phase, and 14 in the transfer phase for the final meta-analysis.

**Figure 4:**
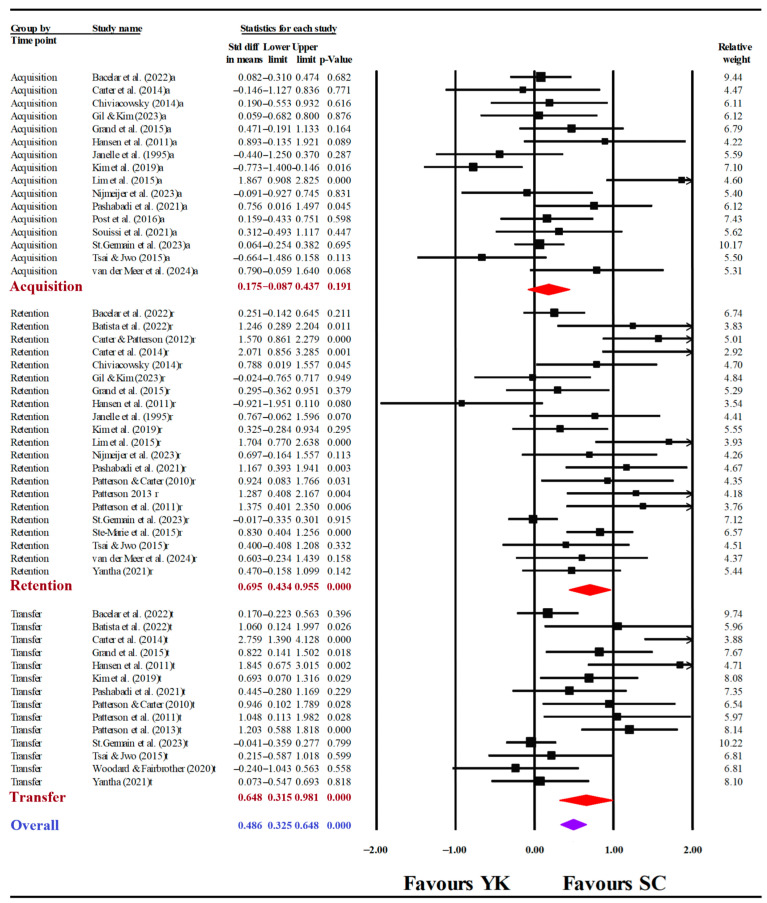
Forest plot of the self-controlled (SC) group compared to the yoked (YK) group. Black squares represent effect sizes from individual studies; red diamonds indicate the pooled effects for the acquisition (a), retention (r), and transfer (t) phases; purple diamonds indicate the overall effect across all phases.

Figure 4 depicts a summary of the random effects meta-analysis and the corresponding forest plot showing SMDs and 95% CIs. The findings were consistent with those of the meta-analysis of SC vs. PR. In the acquisition phase, motor learning performance was similar for the SC and YK groups (SMD = 0.175, 95%CI [−0.087, 0.437], *p* = 0.191). However, the SC groups demonstrated superior motor learning performance compared to the YK groups in both the retention (SMD = 0.695, 95%CI [0.434, 0.955], *p* < 0.001) and transfer (SMD = 0.648, 95%CI [0.315, 0.981], *p* < 0.001) phases.

### 3.6. Moderator Analysis of SC Compared to YK Feedback

The results of the moderator analysis including SMD values, 95% CIs, and homogeneity test statistical values are summarized in Table 3. The following variables were analyzed: feedback model (verbal or visual); environment (outdoor or indoor); skill type (series or discrete), and mean participant age (adult or adolescent). Cognitive status and skill level were not analyzed due to the small sample size (*n* < 3), and for the same reason, environment and mean age were not analyzed in the transfer phase. Moderating variables have different effects on motor skill learning outcomes in different learning phases. The results showed an overall pattern similar to that of the moderator analysis of SC vs. PR.

In the acquisition phase, neither verbal (SMD = 0.092, 95% CI [−0.326, 0.509], *p* = 0.667) nor visual (SMD = 0.234, 95% CI [−0.118, 0.586], *p* = 0.192) feedback; indoor (SMD = 0.155, 95% CI [−0.177, 0.487], *p* = 0.361) nor outdoor (SMD = 0.309, 95% CI [−0.221, 0.839], *p* = 0.254) environments; discrete (SMD = 0.098, 95% CI [−0.077, 0.273], *p* = 0.272) nor series (SMD = 0.630, 95% CI [−1.042, 2.301], *p* = 0.460) skill types; and adolescent (SMD = 0.079, 95% CI [−0.872, 1.029], *p* = 0.871) nor adult (SMD = 0.199, 95% CI [−0.070, 0.467], *p* = 0.147) age groups exerted significant effects.

In the retention phase, significant intervention effects were observed for both verbal (SMD = 0.646, 95% CI [0.401, 0.891], *p* < 0.001) and visual (SMD = 0.763, 95% CI [0.342, 1.184], *p* < 0.001) feedback; for both indoor (SMD = 0.694, 95% CI [0.370, 1.018], *p* < 0.001) and outdoor (SMD = 0.722, 95% CI [0.346, 1.098], *p* < 0.001) environments; series (SMD = 0.793, 95% CI [0.277, 1.310], *p* < 0.01) and discrete (SMD = 0.649, 95% CI [0.311, 0.986], *p* < 0.001) skill types; and for both adolescents (SMD = 0.748, 95% CI [0.329, 1.167], *p* < 0.001) and adults (SMD = 0.690, 95% CI [0.387, 0.993], *p* < 0.001).

In the transfer phase, significant heterogeneity was observed between the two skill type subgroups (Q_(1)_ = 4.435, *p* < 0.05). Specifically, significant intervention effects were observed for both series (SMD = 1.040, 95% CI [0.697, 1.383], *p* < 0.001) and discrete (SMD = 0.478, 95% CI [0.083, 0.873], *p* < 0.05) skill types and for both verbal (SMD = 0.372, 95% CI [0.009, 0.735], *p* < 0.05) and visual (SMD = 0.900, 95% CI [0.381, 1.418], *p* < 0.01) feedback.

## 4. Discussion

We conducted a meta-analysis of 29 quantitative studies to examine the effect of SC feedback in enhancing performance in motor skill learning. Across retention and transfer phases, SC feedback outperformed both PR and YK feedback. However, it did not provide a significant advantage during the acquisition phase, except among cognitively impaired participants. These findings complement earlier work but also highlight the need for careful methodological comparison when evaluating SC feedback’s influence on skill acquisition.

Our results diverge somewhat from Jimenez-Diaz et al. (2020), who reported gains in acquisition that persisted into retention. In contrast, we found SC feedback benefits were more evident in retention and transfer. Key differences include the number of studies (18 vs. 29 through 2024) and the analytic approach: they assessed “remaining” learning from acquisition to retention, whereas we compared performance under different feedback conditions within each phase. Additionally, prior work did not analyze transfer performance, a critical indicator of skill generalization, whereas our meta-analysis found significant advantages for SC feedback. Despite some publication bias, effect sizes remained robust. Our focus was on addressing specific research questions rather than introducing new statistical methods (McKay et al., 2023).

The benefits of SC feedback can be understood within the OPTIMAL (Optimizing Performance through Intrinsic Motivation and Attention for Learning) theory of motor learning (Wulf & Lewthwaite, 2016). According to this framework, motivational and attentional factors enhance motor performance and learning by strengthening the coupling of goals to actions. SC feedback promotes learner autonomy, which enhances performance expectancies and provides a sense of control and competence, increasing interest in the task and self-efficacy (Chiviacowsky et al., 2012a; Ryan & Deci, 2000). In addition, receiving feedback at self-selected times may produce a rewarding effect, triggering dopamine release, which facilitates memory consolidation and skill learning. SC feedback also encourages active engagement with the task, supporting deeper cognitive processing and more effective retention and transfer of skills (Wulf & Lewthwaite, 2016). Together, these mechanisms provide a coherent, theory-based explanation for the observed advantages of SC feedback across retention and transfer phases.

Our meta-analysis corroborates findings that SC feedback’s immediate effects in acquisition can vary depending on feedback frequency. In a few studies, participants receiving SC feedback at a high rate (60–70%) exhibited worse acquisition performance than those in YK conditions (Kim et al., 2019; Tsai & Jwo, 2015). Overreliance on feedback can promote trial-and-error without substantive performance gains, possibly reflecting an arbitrary approach to learning. Although frequent feedback may increase motivation, it can also trigger maladaptive short-term corrections (Schmidt, 1991). Future empirical work is needed to clarify how feedback frequency shapes SC feedback’s influence on skill learning (Hebert & Coker, 2021).

We found that skill type significantly moderated SC feedback’s transfer-phase benefits, with serial tasks showing greater improvement than discrete tasks, particularly when compared to YK. Serial tasks often demand continuous coordination and integration of multiple sub-skills, which may encourage learners to engage more deeply with feedback and apply it more strategically (Patterson & Carter, 2010; Patterson et al., 2011). Engaging with SC feedback under these cognitively demanding conditions may facilitate deeper processing and more flexible learning strategies, thereby supporting stronger transfer performance (Hansen et al., 2011; Kim et al., 2019). Notably, when comparing SC with PR in serial tasks, we observed an effect size in favor of SC feedback in the transfer phase. However, this effect did not reach statistical significance, and thus should be interpreted with caution.

SC feedback’s benefits were especially pronounced among cognitively impaired learners in the acquisition phase. Compared with typically developing peers, these individuals may value feedback opportunities more and engage with them more attentively, thereby extracting greater learning benefits (Zamani et al., 2015). For instance, children with Down syndrome spontaneously adopted a fading feedback schedule to avoid overreliance (Chiviacowsky et al., 2012c). Similarly, SC feedback has been shown to benefit children with ADHD (Pashabadi et al., 2021) and cerebral palsy (Hemayattalab, 2014; Hemayattalab et al., 2013). This tendency to use feedback more deliberately may explain why cognitively impaired learners demonstrated greater gains than healthy participants during the acquisition phase. However, data limitations restricted our moderator analyses largely to the acquisition phase when comparing SC and PR. Further research is needed to solidify our understanding of cognitive status as a moderator.

This study has several limitations. Firstly, many of the included primary studies had relatively small sample sizes, which reduces statistical power, may increase susceptibility to sampling bias, and limits the reliability of the results. Secondly, the majority of participants were college students, restricting the generalizability of the findings to broader populations and age groups. Future research should therefore aim to recruit larger and more diverse samples, ideally through multi-site designs, to enhance statistical power and external validity. Thirdly, although sensitivity analyses suggested the stability of the results, Egger’s tests indicated the presence of publication bias in both the retention and transfer phases, which might have inflated the effect size estimates. Future studies in this field would benefit from strategies to mitigate publication bias, such as preregistration of study protocols, open sharing of data and materials, and greater efforts to publish null findings. Addressing these limitations will not only improve the robustness of the evidence base but will also provide more solid guidance for the practical application of self-controlled feedback in motor learning.

## 5. Conclusions

This meta-analysis highlights the positive impact of self-controlled (SC) feedback on motor skill learning, particularly in terms of retention and transfer. By examining moderating factors such as cognitive state, skill type, and feedback frequency, the findings contribute to a deeper understanding of how SC feedback can be strategically implemented to optimize learning outcomes.

## Figures and Tables

**Table 1:** Main characteristics of included studies.

Study	Feedback Type	Phase	Sample Size	Skill Level	Cognitive Status	Mean Age	Environment	Learning Task	Feedback Model	Number of Trials During Acquisition
Janelle et al. (1995)	YK	A R	36	U	N	19.6	O	Throwing task	Ve	40
Patterson and Carter (2010)	YK	R T	24	U	N	21.4	I	Key-pressing	Vi	90
Ahmadi et al. (2011)	PR	A R	48	N	N	22.3	I	Force production task	Vi	72
Hansen et al. (2011)	YK	A R T	16	U	N	21.8	I	Key-pressing	Vi	80
Patterson et al. (2011)	YK	R T	20	N	N	22.3	I	Key-pressing	Vi	90
Carter and Patterson (2012)	YK	R	40	U	N	45.95	I	Low-friction slide task	Vi	60
Patterson et al. (2013)	YK	R T	48	U	N	21.3	I	Key-pressing	Vi	90
Carter et al. (2014)	YK	A R T	16	U	N	21.35	I	Low-friction slider task	Vi	60
Chiviacowsky (2014)	YK	A R	28	N	N	22.5	I	Coincident-anticipation timing	Ve	30
Hemayattalab (2014)	PR	A R T	15	N	I	12.26	I	Throwing task	Vi	100
Grand et al. (2015)	YK	A R T	36	N	N	23.1	I	Throwing task	Ve	60
Lim et al. (2015)	YK	A R	24	N	N	27.2	I	Taekwondo Poomsae	Vi	72
Ste-Marie et al. (2015)	YK	R	92	N	N	11.1	I	Double-mini trampoline	Ve	60
Tsai and Jwo (2015)	YK	A R T	24	U	N	25.1	I	Hand grip	Ve	60
Zamani et al. (2015)	PR	A R	12	N	I	10	I	Throwing task	Ve	240
Post et al. (2016)	YK	A	44	N	N	21.8	O	Golf putting	Vi	60
Kim et al. (2019)	YK	A R T	42	N	N	16.91	I	Key-pressing	Vi	200
Patterson et al. (2019)	PR	A R	24	U	N	21.9	I	Key-pressing	Vi	80
Woodard and Fairbrother (2020)	YK	T	24	N	N	20.38	I	Continuous tracing task	Ve	32
Hebert and Coker (2021)	PR	A R	95	N	N	21.08	O	Throwing task	Ve	50
Pashabadi et al. (2021)	YK	A R T	30	N	I	9	O	Football chip pass skill	Ve	60
Souissi et al. (2021)	YK	A	24	E	N	10.84	I	Weightlifting snatch	Vi	144
Yantha et al. (2021)	YK	R T	40	N	N	21.77	O	Golf putting	Ve	50
Batista et al. (2022)	YK	R T	20	N	N	24.86	I	Throwing task	Ve	45
Bacelar et al. (2022)	YK	A R T	100	N	N	20.65	I	Throwing task	Vi	100
St Germain et al. (2022)	YK	A R T	152	N	N	20.64	I	Rapid aiming task	Vi	72
Gil and Kim (2023)	YK	A R	28	N	N	22.3	I	Golf putting	Ve	20
Nijmeijer et al. (2023)	YK	A R	22	N	N	22.9	U	Sidestep cutting task	Vi	20
van der Meer et al. (2024)	YK	A R	23	E	N	43.3	O	Tennis	Vi	20

Note: Feedback type: PR—passively received; YK—yoked, YK represents inclusion in both the PR and YK groups, while PR represents inclusion only in the PR group. Phase: A—acquisition; R—retention; T—transfer. Skill level: U—unspecified; N—novice; E—experts. Cognitive status: N—normal; I—impaired. Environment: U—unspecified; I—indoor; O—outdoor. Feedback model: Ve—verbal; Vi—visual.

**Table 2:** Moderator analysis for the SC vs. PR feedback in three phases.

Phase	Moderator	Level	No. of Studies	SMD	95%CI	Homogeneity Test		
						Q	*df*	*p*
Acquisition	Cognitive status *	Normal	18	0.130	[−0.082, 0.343]	4.895	1	0.027
		Impaired	3	0.789 **	[0.246, 1.333]			
	Feedback model	Verbal	8	0.118	[−0.150, 0.387]	0.538	1	0.463
		Visual	13	0.270	[−0.034, 0.575]			
	Environment	Indoor	15	0.205	[−0.076, 0.486]	0.026	1	0.872
		Outdoor	5	0.241	[−0.098, 0.580]			
	Skill type	Series	4	0.446	[−0.705, 1.598]	0.278	1	0.598
		Discrete	15	0.134	[−0.020, 0.287]			
	Mean age	Adult	16	0.176	[−0.030, 0.381]	0.154	1	0.695
		Adolescent	5	0.332	[−0.421, 1.084]			
Retention	Cognitive status	Normal	23	0.535 ***	[0.275, 0.797]	3.373	1	0.066
		Impaired	3	1.768 **	[0.479, 3.056]			
	Feedback model	Verbal	11	0.666 ***	[0.306, 1.025]	0.062	1	0.803
		Visual	15	0.598 **	[0.206, 0.990]			
	Environment	Indoor	20	0.656 ***	[0.321, 0.990]	0.136	1	0.713
		Outdoor	5	0.550 *	[0.098, 1.002]			
	Skill type	Series	8	0.628 *	[0.085, 1.170]	0.001	1	0.995
		Discrete	16	0.630 ***	[0.291, 0.968]			
	Mean age	Adult	21	0.533 ***	[0.246, 0.821]	2.252	1	0.133
		Adolescent	5	1.072 **	[0.430, 1.715]			
Transfer	Feedback model	Verbal	6	0.372 *	[0.009, 0.735]	3.288	1	0.070
		Visual	9	0.941 ***	[0.445, 1.437]			
	Skill type	Series	5	1.040 ***	[0.697, 1.383]	3.334	1	0.068
		Discrete	9	0.553 **	[0.157, 0.948]			
	Mean age	Adult	12	0.719 **	[0.289, 1.066]	0.008	1	0.929
		Adolescent	3	0.704 **	[0.269, 1.140]			

Note: SMD: standardized mean difference; * *p* < 0.05, ** *p* < 0.01, *** *p* < 0.001.

**Table 3:** Moderator analysis for the SC vs. YK feedback in three phases.

Phase	Moderator	Level	No. of Studies	SMD	95%CI	Homogeneity Test		
						Q	*df*	*p*
Acquisition	Feedback model	Verbal	6	0.092	[−0.326, 0.509]	0.261	1	0.609
		Visual	10	0.234	[−0.118, 0.586]			
	Environment	Indoor	11	0.155	[−0.177, 0.487]	0.233	1	0.630
		Outdoor	4	0.309	[−0.221, 0.839]			
	Skill type	Series	3	0.630	[−1.042, 2.301]	0.385	1	0.535
		Discrete	11	0.098	[−0.077, 0.273]			
	Mean age	Adult	13	0.199	[−0.070, 0.467]	0.057	1	0.812
		Adolescent	3	0.079	[−0.872, 1.029]			
Retention	Feedback model	Verbal	9	0.646 ***	[0.401, 0.891]	0.223	1	0.637
		Visual	12	0.763 ***	[0.342, 1.184]			
	Environment	Indoor	16	0.694 ***	[0.370, 1.018]	0.012	1	0.912
		Outdoor	4	0.722 ***	[0.346, 1.098]			
	Skill type	Series	7	0.793 **	[0.277, 1.310]	0.211	1	0.646
		Discrete	12	0.649 ***	[0.311, 0.986]			
	Mean age	Adult	18	0.690 ***	[0.387, 0.993]	0.048	1	0.827
		Adolescent	3	0.748 ***	[0.329, 1.167]			
Transfer	Feedback model	Verbal	6	0.372 *	[0.009, 0.735]	2.670	1	0.102
		Visual	8	0.900 ***	[0.381, 1.418]			
	Skill type *	Series	5	1.040 ***	[0.697, 1.383]	4.435	1	0.035
		Discrete	8	0.478 *	[0.083, 0.873]			

Note: SMD: standardized mean difference; * *p* < 0.05, ** *p* < 0.01, *** *p* < 0.001.

## Data Availability

The datasets used and/or analyzed during the current study are available from the corresponding author on reasonable request.

## Supplementary Materials

The following supporting information can be downloaded at: https://www.mdpi.com/article/10.3390/bs15091291/s1, Supplementary File S1: Excluded studies with exclusion reason; Supplementary File S2: Overall I values after removal of single studies.

## Abbreviations

The following abbreviations are used in this manuscript:

SC	Self-Controlled
PR	Passively Received
YK	Yoked
PRISMA	Preferred Reporting Items for Systematic Reviews and Meta-Analysis
SMD	Standardized Mean Difference
