# Supplementary material for: Self-Controlled Feedback and Behavioral Outcomes in Motor Skill Learning: A Meta-Analysis
Source: Behav Sci (Basel). 2025 Sep 22;15(9):1291. doi: 10.3390/bs15091291 (PMC12467369; doi:10.3390/bs15091291)
Supplement: Supplementary file 1 [file behavsci-15-01291-s001.zip › Supplementary File S1. Excluded studies with exclusion reason.docx.pdf]

Supplementary File S1. Excluded studies with exclusion reason.

Reference	Reason
Aiken, C. A., Fairbrother, J. T., & Alami, A. (2012). The effects of self-controlled video feedback on the basketball set shot. *Journal of Sport & Exercise Psychology, 34*, S64–S64.	2
Aiken, C. A., Fairbrother, J. T., & Post, P. G. (2012). The effects of self-controlled video feedback on the learning of the basketball set shot. *Frontiers in Psychology, 3*.	3
Ali, A., Fawver, B., Kim, J., Fairbrother, J., & Janelle, C. M. (2012). Too much of a good thing: Random practice scheduling and self-control of feedback lead to unique but not additive learning benefits. *Frontiers in Psychology, 3*.	3
Andrieux, M., Boutin, A., & Thon, B. (2015). Self-control of task difficulty during early practice promotes motor skill learning. *Journal of Motor Behavior, 48*(1), 57–65.	3
Avenanti, A., Ossmy, O., & Mukamel, R. (2017). Short-term motor-skill acquisition improves with size of self-controlled virtual hands. *PLoS One, 12*(1).	3
Banos, J. A. C., Tran, A. Q., Aisner, T., & Salvadora, L. T. (2015). Effects of self-controlled feedback on the acquisition of a balance task. *Journal of Sport & Exercise Psychology, 37*, S28–S28.	2
Barros, J. A. C., Yantha, Z. D., Carter, M. J., Hussien, J., & Ste-Marie, D. M. (2019). Examining the impact of error estimation on the effects of self-controlled feedback. *Human Movement Science, 63*, 182–198.	3
Carter, M. J., & Ste-Marie, D. M. (2016). An interpolated activity during the knowledge-of-results delay interval eliminates the learning advantages of self-controlled feedback schedules. *Psychological Research, 81*(2), 399–406.	3
Carter, M. J., & Ste-Marie, D. M. (2017). Not all choices are created equal: Task-relevant choices enhance motor learning compared to task-irrelevant choices. *Psychonomic Bulletin & Review, 24*(6), 1879–1888.	3
Carter, M. J., Smith, V., Carlsen, A. N., & Ste-Marie, D. M. (2017). Anodal transcranial direct current stimulation over the primary motor cortex does not enhance the learning benefits of self-controlled feedback schedules. *Psychological Research, 82*(3), 496–506.	3
Chiviacowsky, S., & Wulf, G. (2002). Self-controlled feedback: Does it enhance learning because performers get feedback when they need it? *Research Quarterly for Exercise and Sport, 73*(4), 408–415.	3
Chiviacowsky, S., & Wulf, G. (2005). Self-controlled feedback is effective if it is based on the learner's performance. *Research Quarterly for Exercise and Sport, 76*(1), 42–48.	3
Chiviacowsky, S., & Wulf, G. (2012). Self-controlled feedback: The importance of confirming good performance. *Journal of Sport & Exercise Psychology, 34*, S78–S78.	2
Chiviacowsky, S., de Medeiros, F. L., Kaefer, A., Wally, R., & Wulf, G. (2008). Self-controlled feedback in 10-year-old children. *Research Quarterly for Exercise and Sport, 79*(1), 122–127.	3
Chiviacowsky, S., Wulf, G., & Lewthwaite, R. (2012). Self-controlled learning: The importance of protecting perceptions of competence. *Frontiers in Psychology, 3*.	3
Chiviacowsky, S., Wulf, G., & Zachry, T. (2004). Self-controlled feedback is effective if based on the learner's performance. *Journal of Sport & Exercise Psychology, 26*, S55–S55.	2

Chiviacowsky, S., Wulf, G., de Medeiros, F. L., Kaefer, A., & Tani, G. (2008). Learning benefits of self-controlled knowledge of results in 10-year-old children. *Research Quarterly for Exercise and Sport, 79*(3), 405–410.	3
Couvillion, K. F., Bass, A. D., & Fairbrother, J. T. (2019). Increased cognitive load during acquisition of a continuous task eliminates the learning effects of self-controlled knowledge of results. *Journal of Sports Sciences, 38*(1), 94–99.	3
Davis, J., Hendrick, J. L., McGinnis, P. M., & Buckenmeyer, P. J. (2011). Effects of self-controlled feedback on learning the squat. *Journal of Sport & Exercise Psychology, 33*, S66–S67.	2
Dong-Youn, L. (2022). Changes in golf swing learning training using positive or negative self-control feedback. *Journal of Sport & Exercise Psychology, 44*, S35–S35.	2
Fairbrother, J. T., Laughlin, D. D., & Nguyen, T. V. (2012). Self-controlled feedback facilitates motor learning in both high and low activity individuals. *Frontiers in Psychology, 3*.	3
Fairbrother, J. T., Meisterjahn, R. J., & Jensen, P. R. (2010). The effects of self-controlled feedback and error estimation on motor skill learning. *Journal of Sport & Exercise Psychology, 32*, S76–S76.	2
Fairbrother, J., Bass, A., von Lindern, A., & Couvillon, K. (2018). Different degrees of choice influence self-controlled feedback effects on motor learning. *Journal of Sport & Exercise Psychology, 40*, S49–S49.	2
Ferreira, B. P., Malloy-Diniz, L. F., Parma, J. O., Nogueira, N., Apolinário-Souza, T., Ugrinowitsch, H., & Lage, G. M. (2019). Self-controlled feedback and learner impulsivity in sequential motor learning. *Perceptual and Motor Skills, 126*(1), 157–179.	3
Figueiredo, L. S., Ugrinowitsch, H., Freire, A. B., Shea, J. B., & Benda, R. N. (2018). External control of knowledge of results: Learner involvement enhances motor skill transfer. *Perceptual and Motor Skills, 125*(2), 400–416.	3
Fischman, M. G. (2015). On the continuing problem of inappropriate learning measures: Comment on Wulf et al. (2014) and Wulf et al. (2015). *Human Movement Science, 42*, 225–231.	3
Fu, H. J., Liu, J., & Sheu, F. R. (2010). Self-controlled performance feedback and learning of an open motor skill. *Research Quarterly for Exercise and Sport, 81*(1), 37–37.	2
Ghanati, H. A., Letafatkar, A., Shojaedin, S., Hadadnezhad, M., & Schöllhorn, W. I. (2022). Comparing the effects of differential learning, self-controlled feedback, and external focus of attention training on biomechanical risk factors of anterior cruciate ligament (ACL) in athletes: A randomized controlled trial. *International Journal of Environmental Research and Public Health, 19*(16).	3
Gonin, M., Hatfield, B., & Shea, J. B. (2017). Self-controlled feedback: When and why subjects ask for feedback. *Journal of Sport & Exercise Psychology, 39*, S133–S133.	2
Goudini, R., Ashrafpoornavaee, S., & Farsi, A. (2019). The effects of self-controlled and instructor-controlled feedback on motor learning and intrinsic motivation among novice adolescent taekwondo players. *Acta Gymnica, 49*(1), 33–39.	3
Hemayattalab, R., Arabameri, E., Pourazar, M., Ardakani, M. D., & Kashefi, M. (2013). Effects of self-controlled feedback on learning of a throwing task in children with spastic hemiplegic cerebral palsy. *Research in Developmental Disabilities, 34*(9), 2884–2889.	3

Huet, M., Camachon, C., Fernandez, L., Jacobs, D. M., & Montagne, G. (2009). Self-controlled concurrent feedback and the education of attention towards perceptual invariants. *Human Movement Science, 28*(4), 450–467.	3
Jaitner, D., & Mess, F. (2019). Participation can make a difference to be competitive in sports: A systematic review on the relation between complex motor development and self-controlled learning settings. *International Journal of Sports Science & Coaching, 14*(2), 255–269.	1
Jalalvand, M., Bahram, A., Daneshfar, A., & Arsham, S. (2019). The effect of gradual self-control of task difficulty and feedback on learning golf putting. *Research Quarterly for Exercise and Sport, 90*(4), 429–439.	3
Janelle, C. M., Barba, D. A., Frehlich, S. G., Tennant, L. K., & Cauraugh, J. H. (1997). Maximizing performance feedback effectiveness through videotape replay and a self-controlled learning environment. *Research Quarterly for Exercise and Sport, 68*(4), 269–279.	3
Januário, M. S., Figueiredo, L. S., Portes, L. L., & Benda, R. N. (2019). Effects of self-controlled knowledge of results on learning a taekwondo serial skill. *Perceptual and Motor Skills, 126*(6), 1178–1194.	3
Jaquess, K. J., Lu, Y., Ginsberg, A., Kahl, S., Lu, C., Ritland, B., … Hatfield, B. D. (2020). Effect of self-controlled practice on neuro-cortical dynamics during the processing of visual performance feedback. *Journal of Motor Behavior, 53*(5), 632–643.	3
Jaquess, K. J., Lu, Y., Iso-Ahola, S. E., Zhang, J., Gentili, R. J., & Hatfield, B. D. (2019). Self-controlled practice to achieve neuro-cognitive engagement: Underlying brain processes to enhance cognitive-motor learning and performance. *Journal of Motor Behavior, 52*(5), 544–557.	3
Jaquess, K., Lu, Y. Z., Ginsberg, A., Lu, C., Ritland, B., Oh, H., … Hatfield, B. (2019). Effective processing of performance feedback during self-controlled practice. *Journal of Sport & Exercise Psychology, 41*, S35–S35.	2
Jimenez-Diaz, J., Chaves-Castro, K., & Morera-Castro, M. (2021). Effect of self-controlled and regulated feedback on motor skill performance and learning: A meta-analytic study. *Journal of Motor Behavior, 53*(3), 385–398.	1
Kaefer, A., Chiviacowsky, S., Meira, C. d. M., & Tani, G. (2014). Self-controlled practice enhances motor learning in introverts and extroverts. *Research Quarterly for Exercise and Sport, 85*(2), 226–233.	3
Khojasteh Moghani, M., Zeidabadi, R., Shahabi Kaseb, M. R., & Bahreini Borujeni, I. (2021). Mental fatigue reduces the benefits of self-controlled feedback on learning a force production task. *Perceptual and Motor Skills, 128*(5), 2398–2414.	3
Kok, M., Komen, A., van Capelleveen, L., & van der Kamp, J. (2019). The effects of self-controlled video feedback on motor learning and self-efficacy in a physical education setting: An exploratory study on the shot-put. *Physical Education and Sport Pedagogy, 25*(1), 49–66.	3
Laughlin, D. D., Fairbrother, J. T., Alami, A., & Post, P. G. (2011). The effects of self-controlled feedback compared to yoked and reduced frequency schedules. *Journal of Sport & Exercise Psychology, 33*, S87–S87.	2
Lemos, A., Wulf, G., Lewthwaite, R., & Chiviacowsky, S. (2017). Autonomy support enhances performance expectancies, positive affect, and motor learning. *Psychology of Sport and Exercise, 31*, 28–34.	3

Makki, R., Abdoshahi, M., & Ghorbani, S. (2021). Effects of self-controlled knowledge of performance on motor learning and self-efficacy: A kinematic study. *Biomedical Human Kinetics, 13*(1), 187–196.	3
McNevin, N. H., Wulf, G., & Carlson, C. (2000). Effects of attentional focus, self-control, and dyad training on motor learning: Implications for physical rehabilitation. *Physical Therapy, 80*(4), 373–385.	1
McRae, M., Patterson, J. T., & Hansen, S. (2015). Examining the preferred self-controlled KR schedules of learners and peers during motor skill learning. *Journal of Motor Behavior, 47*(6), 527–534.	3
Model Demonstration on Learning a Complex Gymnastic Routine. (2021). *Acta Kinesiologica, S1*.	3
Moghani, M. K., Zeidabadi, R., Kaseb, M. R. S., & Borujeni, I. B. (2021). Mental fatigue reduces the benefits of self-controlled feedback on learning a force production task. *Perceptual and Motor Skills, 128*(5), 2398–2414.	3
Munro, A., & Herrington, L. (2014). The effect of videotape augmented feedback on drop jump landing strategy: Implications for anterior cruciate ligament and patellofemoral joint injury prevention. *The Knee, 21*(5), 891–895.	3
Nunes, M. E. d. S., Correa, U. C., Souza, M. G. T. X. d., Basso, L., Coelho, D. B., & Santos, S. (2019). No improvement on the learning of golf putting by older persons with self-controlled knowledge of performance. *Journal of Aging and Physical Activity, 27*(3), 300–308.	3
Patterson, J. T., & Lee, T. D. (2010). Self-regulated frequency of augmented information in skill learning. *Canadian Journal of Experimental Psychology / Revue Canadienne de Psychologie Expérimentale, 64*(1), 33–40.	3
Profeta, V. L. S., Benda, R. N., Leite, C. M. F., Miguel, A. H., & Ugrinowitsch, H. (2010). The effects of self-controlled feedback on participant motivation and motor skill learning. *Journal of Sport & Exercise Psychology, 32*, S120–S121.	2
Sadowski, J., Niźnikowski, T., Mastalerz, A., Łuba-Arnista, W., & Biegajło, M. (2021). The effect of self-controlled and experimenter-controlled frequency of model demonstration on learning a complex gymnastic routine. *Acta Kinesiologica, S1*.	3
Sanli, E. A., Patterson, J. T., Bray, S. R., & Lee, T. D. (2013). Understanding self-controlled motor learning protocols through the self-determination theory. *Frontiers in Psychology, 3*.	1
St. Germain, L., Lelievre, N., & Ste-Marie, D. M. (2019). Variations in observation frequency in a self-controlled learning environment do not modulate learning of a pirouette en dehors. *Journal of Sports Sciences, 37*(18), 2106–2113.	3
Ste-Marie, D. M., Vertes, K. A., Law, B., & Rymal, A. M. (2013). Learner-controlled self-observation is advantageous for motor skill acquisition. *Frontiers in Psychology, 3*.	3
van der Veer, I. P. A., Verbecque, E., Rameckers, E. A. A., Bastiaenen, C. H. G., & Klingels, K. (2022). How can instructions and feedback with external focus be shaped to enhance motor learning in children? A systematic review. *PLoS One, 17*(8).	1
van Maarseveen, M. J. J., Oudejans, R. R. D., & Savelsbergh, G. J. P. (2018). Self-controlled video feedback on tactical skills for soccer teams results in more active involvement of players. *Human Movement Science, 57*, 194–204.	3
van Vliet, P. M., & Wulf, G. (2006). Extrinsic feedback for motor learning after stroke: What is the evidence? *Disability and Rehabilitation, 28*(13–14), 831–840.	1

von Lindern, A. D., & Fairbrother, J. T. (2022). Reduction of feedback availability limits self-control effects. *Frontiers in Sports and Active Living, 4*.	3
von Lindern, A., Bass, A., & Fairbrother, J. (2018). Self-control effects during a reduction of feedback availability. *Journal of Sport & Exercise Psychology, 40*, S69–S69.	2
Williams, C. K., Tseung, V., & Carnahan, H. (2017). Self-control of haptic assistance for motor learning: Influences of frequency and opinion of utility. *Frontiers in Psychology, 8*.	3
Wu, W. F. W., & Magill, R. A. (2011). Allowing learners to choose. *Research Quarterly for Exercise and Sport, 82*(3), 449–457.	3
Wulf, G. (2007). Self-controlled practice enhances motor learning: Implications for physiotherapy. *Physiotherapy, 93*(2), 96–101.	3
Wulf, G., & Toole, T. (1999). Physical assistance devices in complex motor skill learning: Benefits of a self-controlled practice schedule. *Research Quarterly for Exercise and Sport, 70*(3), 265–272.	3
Wulf, G., Raupach, M., & Pfeiffer, F. (2005). Self-controlled observational practice enhances learning. *Research Quarterly for Exercise and Sport, 76*(1), 107–111.	3
Wulf, G., Shea, C., & Lewthwaite, R. (2010). Motor skill learning and performance: A review of influential factors. *Medical Education, 44*(1), 75–84.	1
Ziv, G., Lidor, R., & Levin, O. (2022). Providing choice of feedback affects perceived choice but does not affect performance. *PeerJ, 10*.	3
Shahbaz, R., Saemi, E., Doustan, M., Hogg, J. A., & Diekfuss, J. A. (2024). The effect of a visual illusion and self-controlled practice on motor learning in children at risk for developmental coordination disorder. *Scientific Reports, 14*(1), Article 12414.	3
Drews, R., Pacheco, M. M., Bastos, F. H., & Tani, G. (2024). Self-controlled feedback in motor learning: The effects depend on the frequency of request. *Journal of Motor Behavior, 56*(5), 555–567.	3

Note: 1. Literature review (n = 7). 2. Meeting abstract (n = 14). 3. Not enough data feedback (n = 51).
