# Supplementary material for: Self-Controlled Feedback and Behavioral Outcomes in Motor Skill Learning: A Meta-Analysis
Source: Behav Sci (Basel). 2025 Sep 22;15(9):1291. doi: 10.3390/bs15091291 (PMC12467369; doi:10.3390/bs15091291)
Supplement: Supplementary file 1 [file behavsci-15-01291-s001.zip › Supplementary File S2. Overall I values after removal of single studies.pdf]

Supplementary File S2A. Overall I^2 values after removal of single studies (Combined SC versus PR group).

Phase	Study	I^2
Acquisition (overall I^2 = 49.178)	Janelle 1995	50.812
	Ahmadi 2011	51.507
	Hansen 2011	49.040
	Carter 2014	51.292
	Chiviacowsky 2014	51.702
	Hemayattalab 2014	45.293
	Grand 2015	50.500
	Lim 2015	28.856
	Tsai 2015	46.497
	Zamani 2015	51.659
	Post 2016	51.717
	Kim 2019	38.065
	Patterson 2019	51.467
	Hebert 2021	51.279
	Pashabadi 2021	48.152
	Souissi 2021	51.511
	Gil 2023	51.653
	Nijmeijer 2023	51.328
	Bacelar 2022	51.577
	St.Germain 2023	51.329
	van der Meer 2024	48.760
Retention (overall I^2 = 73.446)	Ahmadi 2011	71.635
	Batista 2022	73.738
	Carter 2012	71.455
	Carter 2014	72.464
	Chiviacowsky 2014	74.289
	Gil 2023	74.078
	Grand 2015	74.455
	Hansen 2011	72.519
	Hebert 2021	73.036
	Hemayattalab 2014	74.146
	Janelle 1995	74.193
	Kim 2019	74.469
	Lim 2015	72.413
	Nijmeijer 2023	74.415
	Pashabadi 2021	73.533
	Patterson 2010	74.153

	Patterson 2011	73.498
	Patterson 2013	73.485
	Patterson 2019	72.889
	Ste-Marie 2015	73.521
	Tsai 2015	74.506
	Yantha 2021	74.506
	Zamani 2015	70.623
	Bacelar 2022	74.235
	St.Germain 2023	71.634
	van der Meer 2024	74.469
**Transfer (overall $I^2 = 69.974$)**	Patterson 2010	71.208
	Hansen 2011	68.231
	Patterson 2011	71.054
	Patterson 2013	67.609
	Carter 2014	63.252
	Hemayattalab 2014	70.409
	Grand 2015	71.300
	Tsai 2015	71.940
	Kim 2019	71.681
	Woodard 2020	70.327
	Pashabadi 2021	72.118
	Yantha 2021	71.257
	Batista 2022	71.012
	Bacelar 2022	70.812
	St.Germain 2023	62.822

Supplementary File S2B. Overall I² values after removal of single studies (Combined SC versus YK group)

Phase	Study	I^2
Acquisition (overall $I^2 = 57.541$)	Carter 2014	60.027
	Chiviacowsky 2014	60.339
	Gil 2023	60.334
	Grand 2015	59.100
	Hansen 2011	57.758
	Janelle 1995	58.048
	Kim 2019	47.906
	Lim 2015	37.285
	Nijmeijer 2023	60.066
	Pashabadi 2021	56.819
	Post 2016	60.358
	Souissi 2021	60.133
	Tsai 2015	55.740
	Bacelar 2022	60.304
	St.Germain 2023	60.144
	van der Meer 2024	57.450
Retention (overall $I^2 = 65.984$)	Batista 2022	66.463
	Gil 2023	66.367
	Grand 2015	67.375
	Hansen 2011	62.700
	Janelle 1995	67.519
	Kim 2019	67.406
	Lim 2015	63.935
	Nijmeijer 2023	67.612
	Pashabadi 2021	66.180
	Carter 2012	62.250
	Patterson 2010	67.223
	Patterson 2011	66.015
	Patterson 2013	66.034
	Ste-Marie 2015	66.540
	Tsai 2015	67.620
	Yantha 2021	67.658
	Carter 2014	63.880
	Chiviacowsky 2014	67.454
	Bacelar 2022	66.334
	St.Germain 2023	56.942
	van der Meer 2024	67.672

Transfer (overall $I^2 = 70.409$)	Batista 2022	71.451
	Carter 2014	63.056
	Grand 2015	71.737
	Hansen 2011	68.470
	Kim 2019	72.154
	Pashabadi 2021	72.680
	Patterson 2010	71.657
	Patterson 2011	71.498
	Patterson 2013	67.641
	Tsai 2015	72.533
	Woodard 2020	70.931
	Yantha 2021	71.188
	Bacelar 2022	71.528
	St.Germain 2023	63.814
